# Potential specific immunological indicators for stroke associated infection are partly modulated by sympathetic pathway activation

**DOI:** 10.18632/oncotarget.10497

**Published:** 2016-07-08

**Authors:** Huan Wang, Fu-Ling Yan, Michael Cunningham, Qi-Wen Deng, Lei Zuo, Fang-Lan Xing, Lu-Hang Shi, Shan-Shan Hu, Ya Huang

**Affiliations:** ^1^ Neurologic Department, Affiliated Zhongda Hospital, School of Medicine, Southeast University, Nanjing, China; ^2^ School of Medicine, Southeast University, Nanjing, China; ^3^ Public Health School of Southeast University, Nanjing, China

**Keywords:** stroke, infection, immune, sympathetic pathway, HLA-DR

## Abstract

**Background:**

Evidence has led to the consideration of immunodepression after stroke as an important contributor to stroke associated infection (SAI). However, so far no specific immunological indicator has been identified for SAI, and the underlying mechanism remains poorly understood.

**Results:**

SAI patients had significantly higher IL-6 and IL-10 levels and lower HLA-DR levels than no-infection patients within 48h after stroke onset. NA significantly increased IL-10 levels, reduced HLA-DR expression, and decreased IL-6 expression by increasing β-arrestin2 expression which reduced the activation of the NF-κB pathway. Propranolol reversed this effect of NA by reducing β-arrestin2 expression.

**Materials and Methods:**

A systematic search for eligible clinical studies was applied to pool the differences in peripheral cytokine levels between infection and no-infection stroke patients. The underlying mechanism behind these differences was investigated in vitro by applying norepinephrine (NA) and lipopolysaccharide (LPS) to simulate sympathetic pathway activation and sepsis respectively in THP-1 cells. Propranolol was applied to determine the effect of reversing the activation of the sympathetic pathway. Immunological indicators were also detected to assess the immune activation of THP-1 cells and measurements of the expression of β-arrestin2, NF-κB, IκBα and phosphor-IκBα were performed to assess the activation of the sympathetic pathway.

**Conclusion:**

IL-6, IL-10 and HLA-DR are good candidate biomarkers for SAI. The activation of the sympathetic pathway could partly account for the specific immunological alterations found in SAI patients including HLA-DR decrease and IL-10 increase, which both could be reversed by propranolol. However, the mechanism underlying IL-6 increase still needs further exploration.

## INTRODUCTION

Stroke associated infections (SAI) have been generally accepted to accompany the acute phase of stroke with an incidence rate of nearly 30-60%, contributing to a poor prognosis and higher mortality in stroke patients [1–3]. Early diagnosis with biomarker(s) as well as targeted treatments is the most effective approaches to avoid these concerns. However, there is still no effective biomarker(s) to predict SAI. Moreover, none of the management strategies, such as prophylactic antibiotic treatment or aspiration prevention, have proven effective for SAI reduction in the clinic [4–5]. Therefore, it is essential to explore its pathogenesis in order to find an effective biomarker(s).

Numerous studies have shown that a profound stroke-triggered immunodepression with significant changes in the levels of inflammatory factors leads to serious infectious complications in stroke patients [6–7]. Both clinical and animal studies show that a multitude of cytokines such as IL-6 and IL-10 are activated after stroke, induce stroke-associated immunodepression, and greatly increase the risk of infection [8–11]. Furthermore, there is increasing evidence that reduced monocytic human leukocyte antigen (HLA-DR) expression can independently predict the occurrence of SAI. Even though it is generally accepted that nuclear factor kappa B (NF-κB) activates the expression of various immunological factors, the mechanism underlying their expression after stroke still remains poorly understood [12–13].

Experimental evidence suggests that the sympathetic pathway is hyperactivated after stroke, which results inimmunodeficiency and an increased susceptibility to SAI [6, 14–15]. Xabier's and Chamorro's clinical studies joined this consensus with their discovery of a strong positive correlation between SAI risk and metanephrine levels [16–17]. Importantly, β-arrestin2, a major molecule in G-protein-coupled receptor signaling and the sympathetic nervous system regulation of the immune system, is able to inhibit NF-κB activation by directly interacting with IκBα [18–19]. NF-κB sequestration also occurs after associating with IκBα under normal conditions [20]. These findings suggest that the activation of the sympathetic β-arrestin2/IκBα/NF-κB pathway might contribute to the increase in expression of immunological factors after stroke.

Hence the purpose of this study was to investigate the specific immunological indicator(s) for SAI and to determine if their expression is promoted by activation of the sympathetic pathway. Furthermore, propranolol, a nonselective beta-blocker, was applied to inhibit the sympathetic pathway activation in order to confirm that the observed effects were due to the sympathetic pathway [21–22].

## RESULTS

### Peripheral cytokines levels were significantly increased in SAI patients

**Figure 1:**
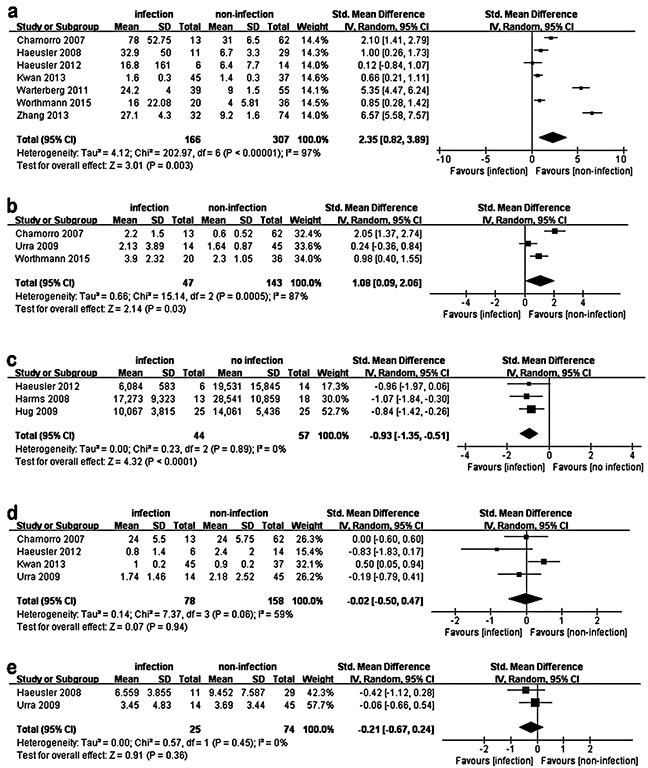
Pooled analysis of the difference in peripheral cytokine levels between SAI patients and no-infection stroke patients The squares and horizontal lines correspond to the study-specific Std.MD and 95%CI. The area of the squares reflects the weight. IV means inverse variance. Because of the significant heterogeneity, a random effects model was used to pool SMD for the included studies. SAI patients had significantly higher IL-6 (**a.** P=0.003) and IL-10 (**b.** P=0.03) levels compared to no-infection stroke patients within 48h after stroke onset. Moreover, a significantly lower HLA-DR level (**c.** P<0.0001) was found in SAI patients. However, TNF-α (**d.** P=0.94) and IFN-γ (**e.** P=0.36) have no statistically significant difference between the two groups.

After a study selection process (Supplemental Data, Supplementary Figure S1), ten articles involving 613 patients were identified for the pooled analyses. The general characteristics of these studies are summarized in Table 1. SAI patients had significantly higher IL-6 (Std.MD 2.35; 95%CI 0.82–3.89; P=0.003) and IL-10 (Std.MD 1.08; 95%CI 0.09–2.06; P=0.03) levels compared to stroke patients without infection within 48h after stroke onset (Figure 1a-b). Moreover, significantly lower HLA-DR levels (Std.MD −0.93; 95%CI −1.35–−0.51; P<0.0001) were found in SAI patients (Figure 1c). However, neither TNF-α (Std.MD −0.02; 95%CI −0.50–0.47; P=0.94) nor IFN-γ (Std.MD −0.21; 95%CI −0.67–0.24; P=0.36) differences were statistically significant (Figure 1d-e). Detailed results of these pooled analyses are further presented in the Supplemental Data.

**Table 1:** Characteristics of included studies

First Author	Publish Year	Study Location	Stroke Patients		Evaluation Indexes	Time of Blood Collection	Type of Article	Quality Score
			Infection	No-infection				
Chamorro [16]	2007	C	13	62	TNF-α, IL-10	Day1	RCT	4
Haeusler [23]	2008	C	11	29	IL-6, IFN-γ	Day1	Pro	6
Urra [17]	2009	C	14	45	IFN-γ, TNF-α, IL-10	Day1	Pro	5
Hug [37]	2009	C	25	25	HLA-DR	Day1	Pro	7
Harms [38]	2008	C	13	18	HLA-DR	Day1	RCT	4
Warterberg [39]	2011	C	39	55	IL-6	Day1	Pro	6
Haeusler [13]	2012	C	6	14	IL-6, TNF-α, HLA-DR	Day1	Pro	7
Zhang [40]	2013	A	32	74	IL-6	Day1	Retro	4
Kwan [8]	2013	C	45	37	IL-6, TNF-α	First 48h	Pro	7
Worthmann [11]	2015	C	20	36	IL-6, IL-10	Day1	Pro	7

The quality of randomized studies was assessed by using the Jadad Scale with 0-3 (low quality) and 4-5 (high quality). The quality of nonrandomized studies was assessed by using the Newcastle-Ottawa Scale (NOS) with 0-5 (low quality) and 6-9 (high quality). C: Caucasian; A: Asian; Pro: prospective; Retro: retrospective.

### Sympathetic neurotransmitter depresses the cytokine levels in THP-1 cells induced by LPS

**Figure 2:**
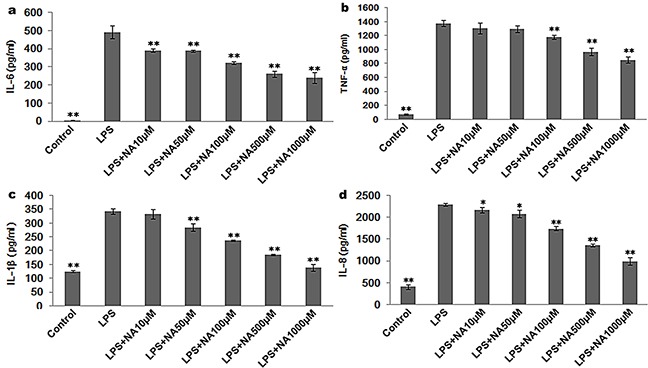
NA stimulation suppresses cytokine release induced by LPS in THP-1 cells **a.** LPS-induced IL-6 was inhibited by NA in a dose-dependent manner. **b.** LPS-induced TNF-α release was decreased by NA at the minimum concentration of 100μM, and a dose-dependent response was observed with concentration increases of 100μM. **c.** LPS-induced IL-1β release was reduced by NA at the minimum concentration of 50μM. A dose-dependent response was observed with concentration increases of 50μM. **d.** LPS-induced IL-8 release was also reduced by NA in a dose-dependent manner. *, P<0.05, and **, P<0.01 compared to the LPS stimulation group.

Compared to THP-1 cells treated with LPS alone, co-treatment with LPS and NA led to a dose-dependent decrease in pro-inflammatory cytokines, with significant decreases in IL-6, IL-8, TNF-α and IL-1β (Figure 2). Interestingly, IL-10, an anti-inflammatory cytokine, was not significantly increased by treatment with LPS and NA. And, NA alone could not significantly increase cytokine release in THP-1 cells (Supplemental Data, Supplementary Figure S2).

In this study there was concern that the ELISA kit might not have sufficient sensitivity to detect IL-10. Although some clinical and animal studies detected IL-10 with ELISA analysis, the levels were much lower than that of other cytokines. For this reason, RT-PCR was applied to detect cytokine expression in the following section.

### Propranolol reverses the immune suppression induced by sympathetic neurotransmitters in THP-1 cells

**Figure 3:**
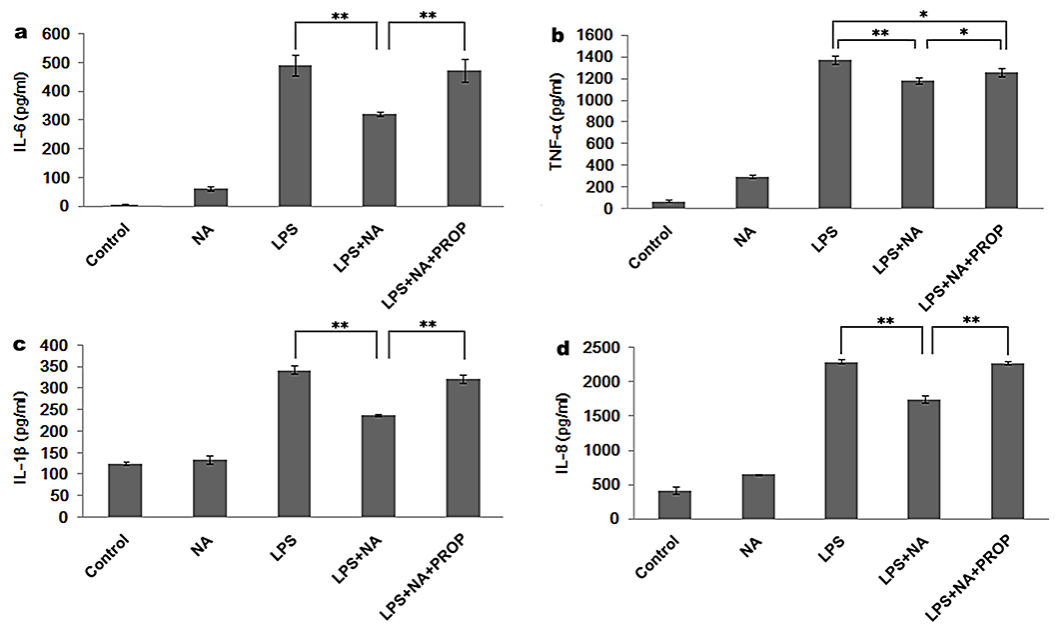
Propranolol reverses the cytokine release modulation of NA in THP-1 cells As each cytokine was significantly suppressed by NA at the concentration of 100μM, 100μM NA was applied in the following study. NA inhibited LPS induced IL-6 (**a**) TNF-α (**b**) IL-1β (**c**) and IL-8 (**d**) release. However, propranolol stimulation could significantly reduce the inhibition effect of NA by increasing the release of IL-6 (a), TNF-α (b), IL-1β (c) and IL-8 (d). *, P < 0.05, and **, P < 0.01.

Considering that the expression levels for each cytokine were strikingly decreased when the cells were treated with NA at the concentrations of 100μM and LPS (P<0.01) (Figure 2), we performed the following experiments using NA at the concentrations of 100μM. As shown in Figure 3, compared to the LPS stimulation group (IL-6: 492.26±35.96pg/ml, TNF-α: 1374.56±42.70pg/ml, IL-1β: 342.45±9.55pg/ml, IL-8: 2292.95±33.35pg/ml), NA co-stimulation significantly inhibited pro-inflammatory cytokines levels induced by LPS in THP-1 cells, including IL-6 (322.11±7.49pg/ml), TNF-α (1181.55±27.91pg/ml), IL-1β (237.60±2.73pg/ml) and IL-8 (1745.88±48.84pg/ml). However, in the presence of propranolol, these cytokine levels climbed substantially (IL-6: 473.83±38.83pg/ml, TNF-α: 1261.56±37.53pg/ml, IL-1β: 322.32±10.02pg/ml and IL-8: 2272.36±26.32pg/ml). For each cytokine level, the difference between the propranolol group and the NA and LPS co-treatment group was statistically significant (P<0.01, P<0.05, P<0.01, P<0.01).

**Figure 4:**
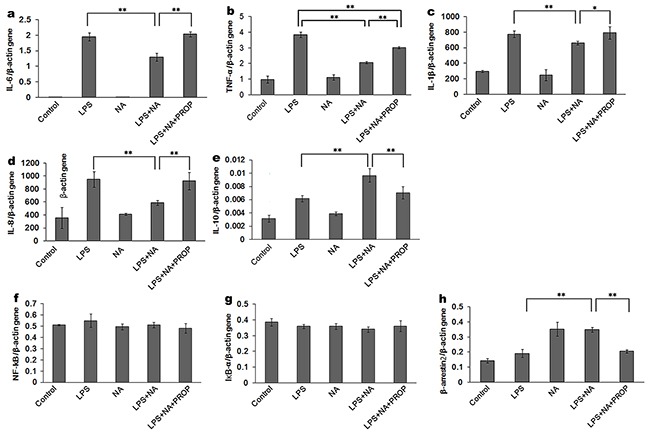
Effect of NA in THP-1 cells on cytokine expression detected at the gene level LPS induced high IL-6 (**a**) TNF-α (**b**) IL-1β (**c**) and IL-8 (**d**) gene expression. With the co-stimulation of NA, the gene expression of these cytokines was significantly decreased (a-d). IL-10 gene expression in the LPS and NA co-stimulation groups were significantly increased compared to the LPS stimulation group (**e**) In the propranolol stimulation group, IL-6 (a), TNF-α (b), IL-1β (c) and IL-8 (d) gene expression were elevated whileIL-10 gene expression was decreased (e). NA stimulation could significantly increase β-arrestin2 gene expression (**h**) NF-κB and IκBα gene expression were not affected in each group (**f-g**) *, P < 0.05, and **, P < 0.01.

The RT-PCR results were consistent with the ELISA analysis showing significantly higher expression of IL-6, TNF-α, IL-1β and IL-8 gene in the propranolol stimulation group compared to the LPS and NA co-stimulation group (P<0.01, P<0.01, P<0.05, P<0.01) (Figure 4a-d). Moreover, as shown in Figure 4e, an increase of IL-10 was detected in the LPS and NA co-stimulation group (0.01±0.001), which was significantly different from IL-10 gene expression in the LPS group (P<0.01). And this expression increase in the LPS and NA co-stimulation group was significantly inhibited by propranolol (0.007±0.001, P<0.01, Figure 4e).

**Figure 5:**
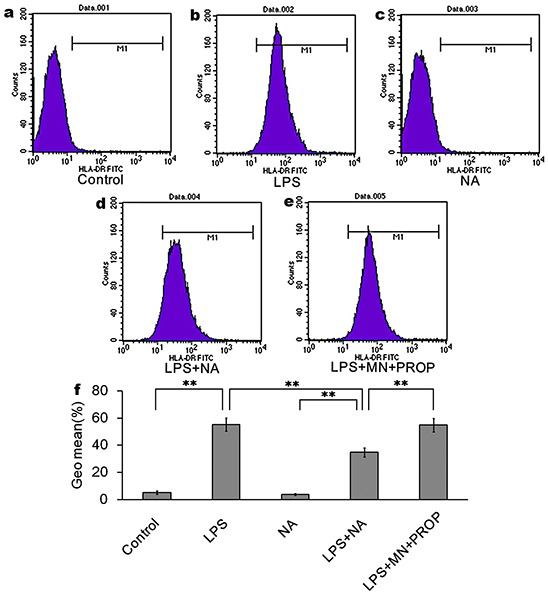
NA inhibits LPS-induced HLA-DR expression, which could be reversed by propranolol Compared to the control group (5.25%±1.27% of cells expressing HLA-DR, (**a**) LPS intervention induced high expression of HLA-DR (55.26%±4.71% of cells expressing HLA-DR, (**b**) NA co-stimulation could significantly inhibit LPS-induced HLA-DR expression (34.83%±3.31%of cells expressing HLA-DR, (**d**) NA stimulation alone did not significantly induce HLA-DR expression (3.75%±0.62% of cells expressing HLA-DR antigen, (**c**) With propranolol stimulation, HLA-DR expression was increased to 54.90%±4.93% of cells (**e**) Data are the mean +/− SD. *, P < 0.05, and **, P < 0.01 (**f**)

The expression of HLA-DR in the LPS group (55.26%±4.71%, Figure 5b) was significantly higher than in the LPS and NA co-stimulation group (34.83%±3.31%, P<0.01, Figure 5d,f). And, propranolol was able to antagonize the suppression effect of NA on HLA-DR expression (54.90%±4.93%, Figure 5e). The difference between HLA-DR expression in the propranolol group and the LPS and NA co-treatment group was statistically significant (P<0.01, Figure 5f).

### Norepinephrine activates the signaling pathway of β-arrestin2/Iκα/NF-κB to restrain immune responses

**Figure 6:**
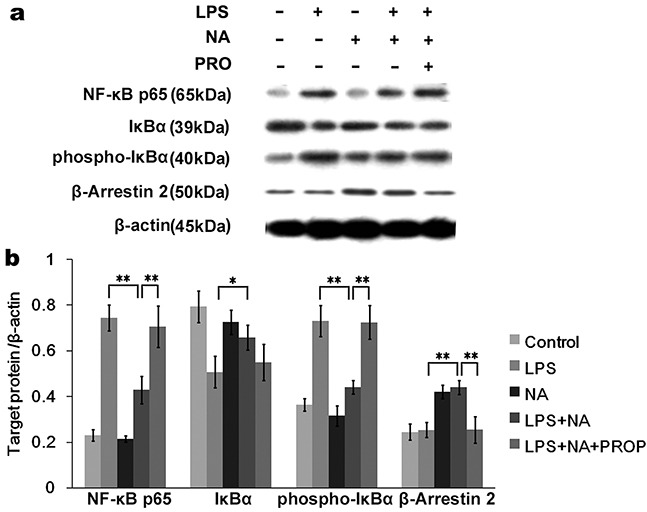
Activated β-arrestin2 by NA inhibits IκBα phosphorylation of NF-κB pathway **a.** The Western blot image showedNF-κBp65, IκBα, phosphor-IκBα and β-arrestin2 expression by THP-1 cells in different groups. **b.** Densitometry analysis shown as graph bars for each protein expression level normalized with β-actin. LPS stimulation significantly activated the NF-κB pathway by increasing phosphor-IκBα and NF-κBp65 expression. With NA co-stimulation, LPS-induced phosphor-IκBα and NF-κBp65 expression were significantly inhibited. Meanwhile, increased β-arrestin2 and IκBα expression were detected. In the propranolol stimulation group, β-arrestin2 was not significantly activated, resulting in increased phosphor-IκBα and NF-κB p65 expression compared to the NA co-stimulation group. *, P < 0.05, and **, P < 0.01.

We further investigated the expression of NF-κB, IκBα and β-arrestin2. As shown in Figure 6, the NF-κB pathway was activated by LPS resulting in increased NF-κBp65 (0.74±0.01), phosphor-IκBα (0.73±0.04) and decreased IκBα (0.51±0.05). In the LPS and NA co-stimulation group β-arrestin2 was significantly increased compared to the LPS group (0.42±0.03, P<0.01), while NF-κB p65 and phosphor-IκBα were decreased (0.21±0.06, P<0.01; 0.32±0.03, P<0.01), and expression of IκBα was increased (0.73±0.05, P<0.05). Propranolol dramatically inhibited β-arrestin2 activation by NA and LPS co-stimulation (0.25±0.06, P<0.01), resulting in increased expression of NF-κB p65 (0.71±0.09, P<0.01) and phospho-IκBα (0.72±0.07, P<0.01).

In contrast to the western blot results for the phosphorylated proteins, PCR analysis did not show a significant difference in the NF-κB and IκBα gene levels (Figure 4f,g) between any of the groups. However, β-arrestin2 gene expression was conspicuously increased by stimulation with NA (Figure 4h). Compared to the LPS and NA co-stimulation group (0.35±0.02), β-arrestin2 gene expression was prominently reduced in the propranolol stimulation group (0.21±0.01, P<0.01).

## DISCUSSION

A growing body of evidence suggests that the immunodepression and immunological alteration preceding SAI after stroke might explain the high incidence of infection in these patients [15]. The identification of specific immunological indicators for predicting SAI remains contentious, even though multiple inflammatory factors have been proposed as possible candidates. Our pooled analysis confirms that IL-6 and IL-10 are significantly increased while HLA-DR expression is dramatically decreased in SAI patients. Moreover, these changes are detectable within 48h after a stroke, which clearly precedes the time window of SAI occurrence [23]. As a result, IL-6, IL-10 and HLA-DR are good candidate biomarkers for the early detection of SAI. Furthermore, exploring the cellular mechanism underlying IL-6, IL-10 and HLA-DR release after stroke could pave the way towards preventing SAI.

Our previous study showed that stroke significantly stimulates sympathetic activation [24]. Moreover, the sympathetic pathway has been reported in multiple experimental studies to be the communication link between the neural and immune systems [15, 25]. Numerous clinical studies have even reported that stroke patients with high NA levels are at increased risk of SAI [16–17]. In this study, we observed that sympathetic pathway activation promotes an anti-inflammatory drive in monocytes by inhibiting the NF-κB pathway phosphorylation activity, involving not only HLA-DR and some pro-inflammatory cytokines inhibition but also increased IL-10 release. Unexpectedly, activation of the sympathetic pathway suppressed LPS-induced IL-6 release, which contradicts our pooled analysis findings of increased peripheral IL-6 levels in SAI patients. These results suggest that activation of the sympathetic pathway partially account for the specific alteration of the peripheral immunological indicators observed in SAI patients.

Research has reported that HLA-DR reduction on monocytesis associated with an increased risk of infection [26]. HLA-DR expression reflects the status of global immune function due to its essential role in the process of antigen presentation [27]. In this study, we confirm that HLA-DR levels can discriminate between SAI patients and no-infection stroke patients before the time window of infection. We also for the first time show how the activation of the sympathetic pathway significantly decreases HLA-DR expression, and demonstrate how this effect can be reversed by propranolol. Accordingly, managing HLA-DR levels and sympathetic pathway activation with treatments such as propranolol in the clinic might be a potential approach for preventing SAI.

Although experimental studies have proven stroke-induced immunodepression by showing decreased peripheral IL-6 production, numerous clinical studies have found that IL-6 expression actually increases after stroke in response to strong inflammatory reactions induced by ischaemic brain injury [28–30]. Our results suggest that very strong inflammatory responses occur in SAI patients which activate anti-inflammatory feedback causing increased IL-10. Previous studies support this connection to immune function by showing that stroke patients with large infarct size had strong immune responses and were more vulnerable to infection [31–32]. As a result, the immunological characteristics of SAI patients could be due to harmful consequences from anti-inflammatory feedback in the peripheral immune system, such as decreased HLA-DR and increased IL-10 levels.

In addition to the sympathetic pathway, the activation of other potential pathways such as the hypothalamic-pituitary-adrenal (HPA) axis and the cholinergic pathway have been reported to mediate dysfunction in peripheral immune cells and increase susceptibility to infection in stroke patients [6, 32–34]. These pathways seem to be important additive mechanisms for the development of SAI. Nevertheless, our findings strongly suggest that inhibition of sympathetic actions is an effective approach for reversing HLA-DR decrease and IL-10 increase in stroke patients, paving the way towards more effective therapies for SAI. Owing to limitations in the number of included studies and available data, the evidence level of the SAI-specific indicators is somewhat limited by publication and selection bias. Hence, large sample and multi-center clinical studies of the specific indicators for SAI are still needed in the future.

## MATERIALS AND METHODS

### Systematic review of clinical studies

A systematic review of clinical studies was performed to demonstrate whether an immunological disorder might be the cause and pathogenesis of SAI by pooling the difference in immunological biomarker(s) levels within three days after stroke between SAI patients and patients without infection. Detailed procedures were made following the guideline of RevMan with the RevMan5.2 software (The Cochrane Collaboration, UK), consisting of a literature search and selection, data extraction, and analyses (Supplemental Data). The standard mean differences (SMD) were calculated to evaluate the differences.

### Cell culture

To investigate the underlying mechanism behind the immunological changes in SAI, human THP-1 monocytes (the Cell Bank of National Academy of Sciences, Shanghai, China) were applied in this study. Cells were cultured in RPMI1640 (Invitrogen) supplemented with 10% heat-inactivated FBS, 100U/mL penicillin and 100mg/mL streptomycin. Before the experimental procedure was performed, THP-1 cells were differentiated into macrophages with PMA (1.28uM, 5*10^5 cells/24 well plate) for 48h. All the cells were incubated at 37°C in a 5% CO_2_ humidified incubator.

### Drug administration

Lipopolysaccharide (LPS) was applied to stimulate THP-1 cells at the concentration of 50ng/ml for 6h to simulate sepsis [35–36]. To better understand the effect of the sympathetic neurotransmitter (norepinephrine, NA, Sigma-I6504, American) on THP-1 cells, dose-response studies were conducted to detect cytokine levels after drug interventions at different concentrations (NA 10μM, 50μM, 100μM, 500μM, 1000μM). Additionally, propranolol (Sigma-P8688, America) was applied to reverse the sympathetic activation.

### Cytokine measurement by ELISA assays

Concentrations of inflammatory cytokines (IL-6, TNF-α, IL-1β, IL-8 and IL-10) secreted by THP-1 macrophages were detected using ELISA kits (JoyeeBiotechnics Co. Ltd, Shanghai, China) according to the manufacturer's instructions.

### HLA-DR detection by flow cytometry

The FITC anti-human HLA-DR was purchased from Biolegend (CA, USA). The expression of HLA-DR was determined by flow cytometry (FACScan, BD Bioscience) according to the manufacturer's instructions. The values were expressed as mean fluorescence intensity (MFI). Data analysis was performed using CellQuest software (BD Bioscience, Franklin Lakes, NJ, USA).

### Protein extraction and western blot analysis

After drug stimulation, THP-1 cells were concentrated and washed three times in cold PBS. Protein extraction was obtained by lysing the cells in 300 μL RIPA lysis buffer with 3 μL PMSF and 3 μL protease inhibitor (Abcam, Cambridge, England). Then, protein concentrations were measured using the BCA protein assay (Beyotime, Shanghai, China). Equal amounts of proteins (20 μg) were electrophoresed in an SDS-PAGE with 8%-10% polyacrylamide gel, and then transferred to a PVDF membrane (Merck Millipore, Billerica, MA, USA). After that, the membranes were blocked with 5% nonfat milk in TBST and then immunoblotted overnight at 4°C with primary antibodies (Table 2). Subsequently, the membranes were washed with TBST three times and incubated with the secondary antibody (Table 2). Finally, the membranes were visualized using chemiluminescence (Amersham, Uppsala, Sweden) and immunoreactivity analysis was performed by an imaging system (Peiqing JS-780, Shanghai, China).

**Table 2:** All antibodies applied in the western blot analysis

Protein	Molecular weight	Source	Company	Antibody concentration
NF-κB p65	65kDa	Rabbit	CST, USA	1:1500
IκBα	39kDa	Rabbit	CST, USA	1:1500
phospho-IκBα	40kDa	Rabbit	CST, USA	1:1000
β-Arrestin 2	50kDa	Rabbit	CST, USA	1:1000
β-actin	45kDa	Rabbit	CST, USA	1:3000
Secondary antibodies	Anti-RabbitlgG(HRP)	Goat	Abcam, UK	1:3000

### RNA extraction and RT-qPCR analysis

After drug stimulation, the total RNA of each group was extracted using the Trizol Reagent (Invitrogen, Carlsbad, CA, USA) following the manufacturer's instructions. Then, complementary DNA synthesis was performed using the Hiscript 1st strand cDNA synthesis kit (Vazyme, Nanjing, China) by PCR (Eppendorf, Hamburg, Germany). Subsequently, RT-qPCR was performed using AceQ™ qPCR SYBR Green Master Mix (Vazyme) in accordance with the manufacturer's protocol on the StepOnePlus™ Real-Time PCR system (ThermoFisher Scientific, USA). Primer sequences for each aim gene are shown in Table 3. The results were expressed as the number of target gene copies per 35 copies.

**Table 3:** All gene primer sequences (Generay Biotechnology, Shanghai, China) applied in the qPCR analysis

Gene (GenBank)		Primer sequence (5′-3′)
IL-6 (NM_000600.3)	Forward	CAGACAGCCACTCACCTC
	Reverse	CTCAAACTCCAAAAGACCAG
IL-10 (NM_000572.2)	Forward	GGAGAACCTGAAGACCCT
	Reverse	TGATGAAGATGTCAAACTCACT
IL-1β (NM_000576.2)	Forward	ACCACCACTACAGCAAGG
	Reverse	AAAGATGAAGGGAAAGAAGG
IL-8(NM_000584.3)	Forward	GCATAAAGACATACTCCAAACC
	Reverse	AAACTTCTCCACAACCCTCT
TNF-α (NM_000594.3)	Forward	TGTAGCAAACCCTCAAGC
	Reverse	GGACCTGGGAGTAGATGAG
NF-κB (NM_001165412.1)	Forward	CCACAAGCAAGAAGCTGAAG
	Reverse	AGATACTATCTGTAAGTGAACC
IκBα (NM_020529.2)	Forward	ACACTAGAAAACTTCAGATGC
	Reverse	ACACAGTCATCATAGGGCAG
ARRB2 (NM_001257331.1)	Forward	TGTGGACACCAACCTCATTG
	Reverse	TCATAGTCGTCATCCTTCATC
β-actin (NM_001101.3)	Forward	GCACCACACCTTCTACAATGAG
	Reverse	ATAGCACAGCCTGGATAGCAAC

### Statistical analysis

Every experiment was performed in triplicate. The results were expressed as means ± standard deviations, and statistically analyzed through one-way analysis of variance (ANOVA) and Student's t-tests. P<0.05 was considered to be statistically significant. All statistical analyses were performed using SPSS 17.0 (Statistical Package for the Social Science, SPSS Ins., IL, USA).

## CONCLUSION

Peripheral IL-6, IL-10 and HLA-DR are good candidate biomarkers for the early detection of SAI. The activation of the sympathetic pathway could partly explain the specific alteration of the immunological characteristics observed in SAI patients, resulting in a decreasing HLA-DR level and increasing IL-10 level. However, the cellular mechanism underlying IL-6 release in SAI patients needs further exploration.

## SUPPLEMENTARY MATERIALS AND METHODS, REFERENCES, FIGURES


