# Supplementary material for: Potential specific immunological indicators for stroke associated infection are partly modulated by sympathetic pathway activation
Source: Oncotarget. 2016 Jul 8;7(32):52404–15. doi: 10.18632/oncotarget.10497 (PMC5239561; doi:10.18632/oncotarget.10497)
Supplement: Supplementary file 1 [file oncotarget-07-52404-s001.pdf]

Potential specific immunological indicators for stroke associated infection are partly modulated by sympathetic pathway activation

SUPPLEMENTARY MATERIALS

MATERIALS AND METHODS

Identification of literature

Systematic electronic searches of the PubMed and Web of Science citation databases were performed from their inception to September 1th, 2015. Six authors performed the data extraction. We also manually searched the conference proceedings of the World Congress of Neurology, the American Academy of Neurology and the European Federation of Neurological Societies from the year 2000–2015 for relevant clinical trials. The reference lists of all relevant studies were also manually searched for this review. The search subject terms were: infection, pneumonia, immune, immunodepression, brain infarction, cerebral infarction, cerebrovascular accident and stroke. The search was restricted to English-language literature.

Inclusion criteria

1. Clinical studies including cross-sectional, case-control, or cohort study were considered to be eligible for the study pool, and the placebo arms presented separately in the original articles were also included. Only the most recent publication results from the same studies were included.

2. The subjects of eligible studies should be ischemic or hemorrhagic stroke patients diagnosed through clinical and radiographic evidence with subgroup analysis of infection.

3. The criteria of infection definition originated in the included studies. Diagnosis of pneumonia required fever, pathological percussion sounds and purulent secretion, and additional infiltrates in radiographic findings. For UTI to be diagnosed, dysuria and a positive urine culture were required. Infection was defined as developing infection after at least 48h of hospitalization [1].

4. The studies permitted assessment of the immunological blood biomarker measurements (eg, IL-6, TNF-α) after stroke in patients with and without infection and identified the population for both groups.

5. Cytokines levels were measured within 48h after stroke onset.

Exclusion criteria

1. Reviews, case reports, case series, in vitro and animal experiments.

2. Treatment arms in randomized controlled trials were excluded due to confounding from drug related changes in the immunological biomarker levels.

3. The objects of studies were not stroke patients.

4. The objects of studies were immunocompromised by chemotherapy or acquired immunodeficiency syndrome.

Quality assessment and data extraction

The randomized controlled studies were assessed in strict accordance with the Jadad scale [2], and the other studies were assessed in terms of the Newcastle-Ottawa Scale (NOS) [3]. The extracted data from each study included the following: first author's surname, publication time, study location, the total number of cerebral infarction patients, the number of patients with infections and the number without infections, Evaluation indexes: the concentration of each immunological blood biomarkers (eg, IL-6, TNF-α). Two reviewers independently carried out data extraction to avoid evaluation deviation. In case of disagreements, the two reviewers discussed in order to reach an accord.

Data analysis and statistical methods

All of the included studies after systematic review were pooled according to their immunological blood biomarkers (eg, IL-6, TNF-α). For studies with median (IQR) or mean (confidence interval) data, we estimated the standard deviation according to the RevMan user guideline [4]. Review Manager 5.2 (RevMan5.2) (The Cochrane Collaboration, UK) was applied to perform all statistical analyses.

Statistical heterogeneity among all included studies was considered to be statistically significant if $P<0.05$ or $I^2>50\%$ by the assessment of Chi^2 test and inconsistency (I^2) statistics [5]. Subsequently, a random effects model would be applied on account of significant heterogeneity. Otherwise, a fixed-effects model was selected. As the data of blood cytokines levels were continuous values with a wide variation among eligible studies, the Standard mean differences (SMD) were calculated to estimate whether blood cytokines were significantly different between the groups of patients with and without infection. Additionally, publication bias was assessed through Egger's test together with Begg's test (STATA 12.0, Stata Corp. College Station, Texas). $P<0.05$ was regarded to be statistically significant.

RESULTS

Eligible studies

As shown in S1, the primary literature search identified 1706 potentially relevant citations. A review of the titles and abstracts eliminated 1550 articles (479 reviews, 928 basic experiments, and 143 citations with no-relevance). The remaining promising 156 articles were investigated further in greater detail through full-text assessment. From 156 eligible articles, ten had all necessary information for inclusion in the meta-analysis, with biomarker(s) levels expressed as mean ± SD or median (IQR).

IL-6

Data for IL-6 was available in seven trials of 473 patients. Because the I^2 statistic in the fixed effects model demonstrated statistically significant heterogeneity (I^2, 96%; P<0.00001) and a wide variation in the IL-6 levels among studies was found, a random effects model was used to pool the Std. mean difference for the included studies. The pooled result demonstrated that SAI patients had a significantly higher IL-6 level than patients without infection (Std.MD 2.35; 95%CI 0.82–3.89; P=0.003).

TNF-α

Four studies (236 patients) were collected to estimate the difference in the TNF-α level between patients with and without infection. Because the I^2 statistic in the fixed effects model demonstrated statistically significant heterogeneity (I^2, 73%; P =0.001) and a wide variation in TNF-α levels among studies was found, a random effects model was used to estimate the difference in the TNF-α levels between the two groups by the pooling the Std. mean difference. Compared to patients without infection, the TNF-α level of patients with infection was a little lower, but the difference was not statistically significant (Std.MD −0.02; 95%CI −0.50–0.47; P = 0.94).

IFN-γ

Two studies with 99 patients detected IFN-γ level in patients and compared the difference between the groups with and without infection. There was no heterogeneity between studies (I^2 = 0%, P=0.40) when IFN-γ levels were compared between patients with and without infection. Since this wide variation in the data was not corrected for potential confounders, we also estimated the difference between the IFN-γ level of the groups with and without infection by pooling the Std. mean difference in a random effects model. Patients with infection had a lower IFN-γ level than those without, but the difference was not statistically significant (Std.MD −0.21; 95%CI −0.67–0.24).

IL-10

Three studies including 190 patients were used to compare the difference in the IL-10 level between the groups with and without infection. Due to the wide variation in the data and the high heterogeneity score for the included studies (I^2 = 78%, P=0.0003), we also estimated the difference in the IL-10 level between the groups by pooling the Std. mean difference in a random effect model. Patients with infection had a significantly higher IL-10 level than patients without infection (Std.MD 1.08; 95%CI 0.09–2.06; P = 0.03).

HLA-DR

The difference in the HLA-DR level between patients with and without infection was evaluated in three studies containing with 101 patients. A random effects model was used to calculate the pool values due to a wide variation in the HLA-DR levels between studies, although significant heterogeneity was not found (P=0.89, I^2 = 0%). Compared to patients without infection, the HLA-DR level in patients with infection was significantly lower (Std.MD −0.93; 95%CI −1.35–−0.51; P <0.001).

Publication bias

The Egger's test for publication bias about IL-6, TNF-α, and IL-10 (t = 1.82, P = 0.128; t = −1.17, P = 0.230;t = −2.88, P = 0.213) suggested that there was no significant publication bias, while the results of IFN-γ and HLA-DR did show publication bias might be present (none data; t=-60.47, P=0.011). The evaluation of publication bias was somewhat limited by the small number of studies identified for inclusion in the pooled analyses.

REFERENCES

1. Haeusler KG, Schmidt WU, Föhring F, Meisel C, Helms T, Jungehulsing GJ, Nolte CH, Schmolke K, Wegner B, Meisel A, Dirnagl U, Villringer A, Volk HD. Cellular immunodepression preceding infectious complications after acute ischemic stroke in humans. Cerebrovasc Dis. 2008; 25:50-8.
2. Jadad AR, Moore RA, Carroll D, Jenkinson C, Reynolds DJ, Gavaghan DJ, McQuay HJ. Assessing the quality of reports of randomized clinical trials: is blinding necessary. Control Clin Trials. 1996; 17: 1-12.
3. Stang A. Critical evaluation of the Newcastle-Ottawa scale for the assessment of the quality of nonrandomized studies in meta-analyses. Eur J Epidemiol. 2010; 25: 603-5.
4. Review Manager. RevMan User Guideline Version 5.2 for Windows. Oxford, England: The Cochrane Collaboration, 2012.
5. Higgins JP, Thompson SG, Deeks JJ, Altman DG. Measuring inconsistency in meta-analyses. BMJ. 2003; 327: 557-60.

SUPPLEMENTARY FIGURES

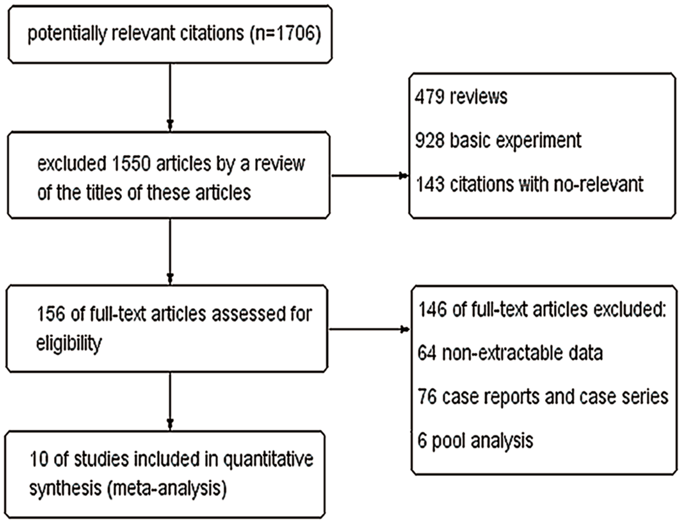

Supplementary Figure S1: Electronic search flow chart.

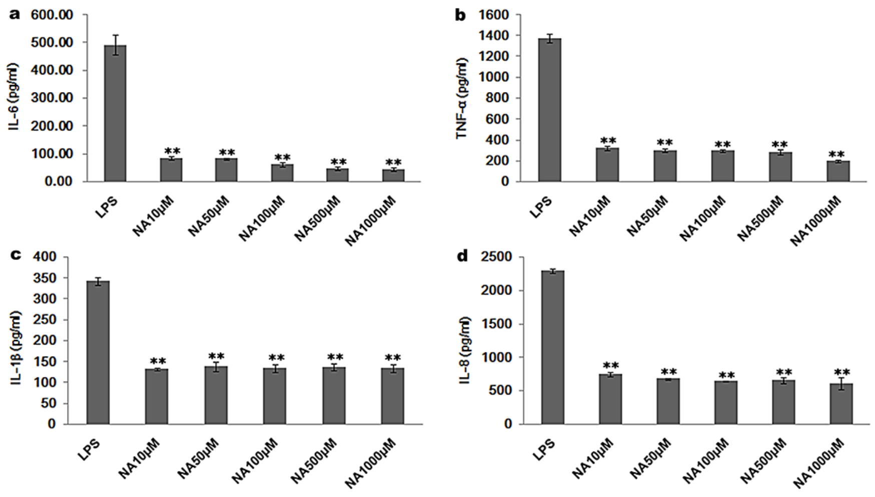

Supplementary Figure S2: NA stimulation alone does not activate THP-1 cells. Cytokines levels were detected to assess the immune activation of THP-1 cells. After the cells were stimulated with NA at different concentrations, supernatant liquid was collected and each cytokine concentration was determined using ELISA kit. With the stimulation of NA at different concentrations, the levels of IL-6 **a.** TNF-α **b.** IL-1β **c.** and IL-8 **d.** were significantly lower than those with LPS stimulation. **, P<0.01 by one-way ANOVA compared with the LPS stimulation group.
